# Systematic development and refinement of a contextually relevant strategy for undergraduate medical ethics education: a qualitative study

**DOI:** 10.1186/s12909-020-02425-6

**Published:** 2021-01-06

**Authors:** Muhammad Shahid Shamim, Adrienne Torda, Lubna A. Baig, Nadeem Zubairi, Chinthaka Balasooriya

**Affiliations:** 1 Dow Institute of Health Professionals Education, Dow University of Health Sciences, Karachi, Pakistan; 2 Scholar at the University of New South Wales, Sydney, Australia; 3 UNSW Medicine, University of New South Wales, Sydney, Australia; 4 APPNA Institute of Public Health, Jinnah Sindh Medical University, Karachi, Pakistan; 5 Lead for Ethics & Professionalism Course at Rabigh Medical College, King Abdulaziz University, Jeddah, Saudi Arabia; 6 Medical Education Development, School of Public Health & Community Medicine, University of New South Wales, Sydney, Australia

**Keywords:** Ethics, Educational strategy, Undergraduate medical education, Workbook

## Abstract

**Background:**

Delivery of medical ethics education is complex due to various reasons, compounded by the context-dependent nature of the content. The scarcity of relevant resources in the contexts of some developing countries adds a further layer of difficulty to ethics education in these contexts. We used a consultative approach with students, teachers and external experts to develop a practical approach to medical ethics education. This study aimed to develop and refine a contextually relevant approach to ethics education in the region of Saudi Arabia.

**Methods:**

The study utilised an explorative qualitative methodology to seek views of students and faculty of Rabigh Faculty of Medicine, Saudi Arabia, and international experts in the field of ethics and education to review and enhance a new ethics learning strategy which included a workbook-based tool. Three focus groups with 12 students, in-depth interviews with four faculty members and qualitative feedback from eleven external experts enabled the study participants to objectively critique the WBEL and provide feedback to enhance its quality. Thematic content analysis of the data was done to draw inferences which were used to refine the educational strategy.

**Results:**

The analysis generated twenty-one sub-themes within four main themes: design features, content, teaching methods and assessment. These findings helped to design the educational strategy to improve its effectiveness in the given context.

**Conclusion:**

The study drew on the views of students, faculty and external experts to systematically develop a novel approach to ethics education for countries like Saudi Arabia. It also demonstrated the use of the consultative approach for informing a culturally relevant educational strategy in the Middle East context.

**Supplementary Information:**

The online version contains supplementary material available at 10.1186/s12909-020-02425-6.

## Background

Medical ethics education is complex for many reasons, including the context-dependent nature of the content. At least part of the difficulty in developing and delivering a medical ethics curriculum lies in the fact that the ethical and moral behaviour of a professional is constructed through the interaction of personal and environmental influences. It is based on obligations and expectations of both the profession and society and is not a universally homogenous phenomenon [1, 2]. This intricate relationship between cultural context and demands of professional conduct, pose challenges to the teaching and assessment of ethics in different regions of the world. This struggle is far more significant in the Middle Eastern countries where, in addition to the cultural differences [3], other factors play their part. For example, the discipline of medical ethics has not developed indigenously in this region. It has been adapted from western countries, mostly the UK and USA, along with the science subjects in curricula. However, unlike science subjects, medical ethics is profoundly entwined with the socio-cultural contexts of respective societies which, in the studied regions, are markedly different from the Western World [4]. Therefore, resources like textbooks and literature, from the West, that serve appropriately for teaching scientific facts may not be as relevant to ethics education in the contexts of countries in the Middle East.

AlKabba et al. [5] present similar views in the context of medical ethics education in Saudi Arabia, where a single religious theology substantially inspires the legal and moral frameworks. They argue that “importation” of a secular western curriculum of medical ethics is “problematic” in Saudi Arabia, as it is not in line with religious teachings, and does not address legal and moral issues from a local contextual perspective [5]. This can compound the complexities for both learners and teachers and pose challenges to planning and delivering medical ethics courses [6–8].

The Saudi Commission for Health Specialties (SCFHS), the medical accrediting body, clearly states in its syllabi documents that medical ethics need to be taught at all medical institutes in the country [9]. However, SCFHS does not guide when and by whom they should be taught, as they guide for basic and clinical disciplines. Local literature is scarce on the subject. Text on ethics, relevant to the local cultural context, is not available for students or teachers. Institutes have incorporated ethics in their formal curriculum with minimal weightage, either as a short regular or elective course. The teaching and assessment processes in these courses are usually not organised like other disciplines [10]. Subject specialists for teaching ethics are not available, and teaching is performed by basic or clinical science faculty with an interest in ethics teaching. These teachers are usually not trained or guided for delivery of ethics education [11]. Didactic lectures are the main form of teaching, and assessment, if conducted, is mostly through MCQs testing recall of knowledge. Hence, a shortage of trained medical ethics teachers and non-availability of contextually relevant text for students to use as learning resources are the key factors hindering the progress of formal ethics education [5, 12]. An educational approach that can guide ethics teachers and provide learning support for the students is a high priority need in the region.

The Bachelor of Medicine and Bachelor of Surgery (MBBS) is a 5-year program in Rabigh Faculty of Medicine (RFM), Saudi Arabia. The medium of medical education is English; however, patient interaction is mostly in the local language. The MBBS programs at RFM is delivered through an integrated modular approach in the first two basic-science years and discipline-based clinical modules in the latter 3 years. Clinical rotations, where students interact with patients, commence at the start of clinical modules. In the RFM curriculum, medical ethics is incorporated as a two-week module early in the clinical year to fulfil the SCFHS requirement.

Previously this module was facilitated by basic-science teachers, as a series of didactic lectures on topics of their choice, without the presence of a documented plan. Feedback on this previous approach was that students considered this module to be of limited value. This feedback prompted us to review possible strategies and tools to improve the delivery of medical ethics teaching at RFM [13]. The aim was to develop a strategy that is student-centred, contextually relevant and can be used effectively within the constraints that limit the delivery of ethics education in the region.

We initially developed a learning tool to deliver content and work on ‘ethical sensitivity skills’ which we called a ‘Workbook’. The preliminary version of the Workbook was a collection of reading material, case vignettes, and reflective exercises on topics related to medical ethics. It was used as a resource for teachers and students, along with other teaching methods, to facilitate learning during the medical ethics module. An initial pilot survey, using quantitative feedback from students of RFM regarding the use of a Workbook for ethics education, revealed encouraging findings [13]. However, the findings also highlighted some areas for improvement.

**Fig. 1:**
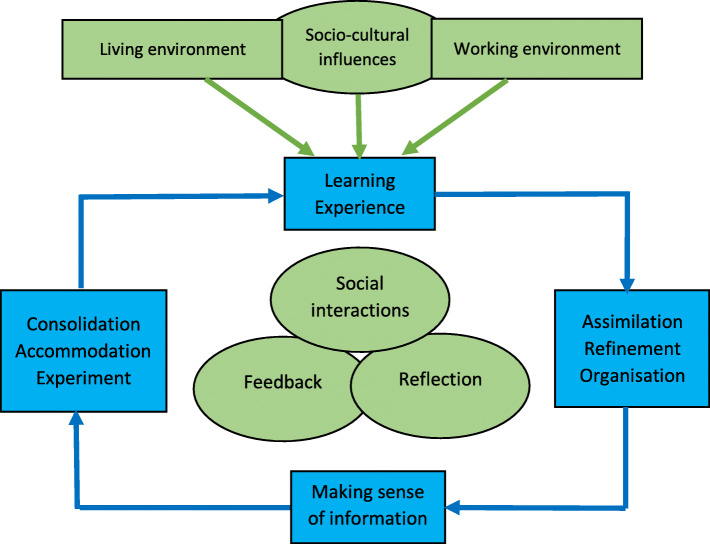
Contextually Relevant Ethics Education Model (CREEM) (M. S. Shamim, 2020)

This feedback became the trigger for conducting an in-depth study to refine and develop a more comprehensive, student-centred approach to ethics teaching, with an updated version of the Workbook as its central component. The resultant approach to teaching, named Workbook-based ethics learning (WBEL) strategy, looks at ethics education through the lens of contextually relevant ethics education model [14]. The model (Fig. 1) incorporates a contextual, relevant task or experience to initiate the process of learning. The learning then takes place through social interactions in the form of discourse, reflective exercises and feedback. The process provides learning opportunities for students to make sense of newly acquired knowledge after which they proceed to apply this knowledge by completing the relevant activities in the Workbook. Reflection and feedback during the learning process enhance students’ motivation and internalisation of knowledge [15]. Social interaction with other students and facilitators during these activities enrich the experience of acquiring and understanding of new information [16]. Thus, the WBEL strategy tends to allow learners to see beyond their personal beliefs within their cultural context and encourages them to engage in discourse that is critical for deeper learning of ethics content.

This study was, therefore, designed to systematically develop, evaluate and refine the preliminary version of the educational strategy through consultative feedback from local and external relevant sources, to enhance its effectiveness for the process of medical ethics education in the context of Saudi Arabia. The consultative feedback method for collecting and correlating data from various sources has been effectively used for educational evaluation in different disciplines during the last two decades [17–20]. This correlation helps in drawing inferences without jeopardising the credibility of research [21, 22]. This paper builds on previous work in this area and describes the development of WBEL as a potentially effective strategy for teaching ethics in undergraduate medical education in the given context.

## Methods used in the research

Epistemologically, this research takes a constructivist view. The data and analysis are created through the researcher’s shared experiences and interactions with participants [23]. In this approach, the values of both the researcher and the research participants contribute to knowledge. In-line with this epistemology, a constructive exploratory approach, using a qualitative method [24, 25] for data collection, was employed for this research. The method was chosen for its ability to facilitate exploration of the WBEL strategy within its context of medical ethics education using consultative feedback from a variety of sources. This approach, therefore, led to exploring a range of perspectives, ensuring a better understanding of multiple facets [26] and enhanced the data credibility [27] through triangulation [28]. The methods and sources for data collection included:

- Focus group discussions with students of RFM who attended the ethics course
- In-depth interviews with faculty members who facilitated the course
- Expert critique through consultation with external experts in the field from various parts of the world

The data sources included a mix of students and faculty, along with external experts familiar with the educational contexts. This enabled an exploration of the many facets of ethics education from a range of socio-cultural and educational angles.

The study was conducted at RFM, which offers a relatively new undergraduate program for male students only. The students at RFM are a mix of conservative and modern Saudi nationals. Faculty members at RFM are mostly medical specialists from various countries in the region (Table 1).

**Table 1:** Characteristics of participating Faculty members and Experts

**Faculty members**			
	**Specialty/ Discipline**	**Nationality**	**Work experience**
1	Pediatrics	Egyptian	Egypt & Saudi Arabia
2	Pediatrics	Pakistani	Pakistan & Saudi Arabia
3	Surgery	Pakistani	Pakistan, Saudi Arabia & UK
4	Family Medicine	Pakistani	Pakistan & Saudi Arabia
**Experts in the field**			
	**Specialty/ Discipline**	**Nationality**	**Work experience**
1	Ethics	Turkish	Turkey
2	Ethics	Turkish	Turkey
3	Education	Saudi	Saudi Arabia
4	Education	Saudi	Multiple countries in the Middle East
5	Education	Egyptian	Egypt & Saudi Arabia
6	Ethics	Philippine	Philippines
7	Ethics	Pakistani	Pakistan & UK
8	Education & Ethics	Pakistani	Pakistan & Kenya
9	Ethics	Pakistani	Pakistan
10	Education	Canadian	Multiple countries in the Middle East & Canada
11	Ethics	Mexican	Mexico

Data were collected during a two-week stand-alone course on medical ethics in the academic year 2017–2018 and included the use of the WBEL.

### Focus group discussions (FGD) and in-depth interviews (IDIs)

The FGD and IDI were conducted by the same researcher (MSS) to ensure homogeneity during data collection, using inquiry protocols explicitly developed for this research [29]. The inquiry protocol contained questions for guiding the FGD and IDIs regarding the main themes. These questions were identified a priori for evaluating the use of Workbook in the ethics course and guided the process for data collection [30].

There was a total of 46 students in year three at RFM who used the Workbook during the medical ethics course. All of them were informed about the study and invited to participate in the FGDs. Twelve students volunteered in groups of 5, 4 and 3, and focus group discussions were conducted with these three groups. The FGD sessions continued for about 30–40 min each.

Five faculty members with interest in teaching ethics facilitated the ethics course. They were invited to participate in the IDIs, and four of them consented to participate. Semi-structured, one-to-one key informant IDIs were conducted with these four faculty members. They belonged to various disciplines (Table 1) with varying teaching experience in the region. Each interview was of approximately 40 min duration.

The researcher who conducted data collection was well known to the participants as a helpful and friendly faculty member and colleague. This relationship may have helped in reducing anxiety in study participants and ensuring data credibility [31, 32], but may also be seen as a limitation (as discussed within the limitations section). The FGDs and IDIs were digitally recorded. During the sessions, the researcher also took notes of the discussed points and leading suggestions by the participants to maintain an audit trail [33]. After each session, the researcher confirmed the noted points and suggestions with the participants. The digital recordings of FGDs and IDIs were transcribed verbatim and de-identified before analysis. Two of the four interviewees confirmed the transcripts through member checking, ensuring the appropriateness of the transcribing process [33]. Thematic content analysis of each transcript was done independently by two reviewers (MSS and NZ).

### Expert critique

The experts in the field of ethics or education from various geographical regions were selected according to the criterion-based purposive sampling technique described by McKenna & Main [34, 35]. The criteria for selecting the experts for this study included:

- Formal qualification and positions in the field of ethics or medical education
- Experience in the field of medical ethics or medical education in the developing country context
- Published in the discipline of ethics education in peer-reviewed journals
- Willingness to participate voluntarily

The researchers (MSS and NZ) identified twenty experts who fulfilled all the selection criteria. These experts were invited to provide consultative feedback on the draft workbook via email. The email included information regarding the primary researcher, study purpose, the process of informed consent and maintaining confidentiality, along with guidelines for critiquing the attached Workbook. Eleven out of twenty experts responded with the requested qualitative feedback. The responses were extracted from their emails, anonymised and saved electronically for thematic content analysis, as for other data sets.

### Ethical considerations

The ethics approval for this study was acquired from the ethics review committee of KAU, reference no. 393–15, where the research was conducted and the University of New South Wales (UNSW), approval no. HC15640, Australia, where the primary author is enrolled as a PhD scholar. The potential participants were informed about the researchers and the study in detail. The Participant Information Statement and Consent Form prescribed by UNSW was used to seek informed written consent from students and faculty participating in the study. All the data were anonymised before analysis.

### Thematic content analysis of focus groups, interviews and expert critique

**Fig. 2:**
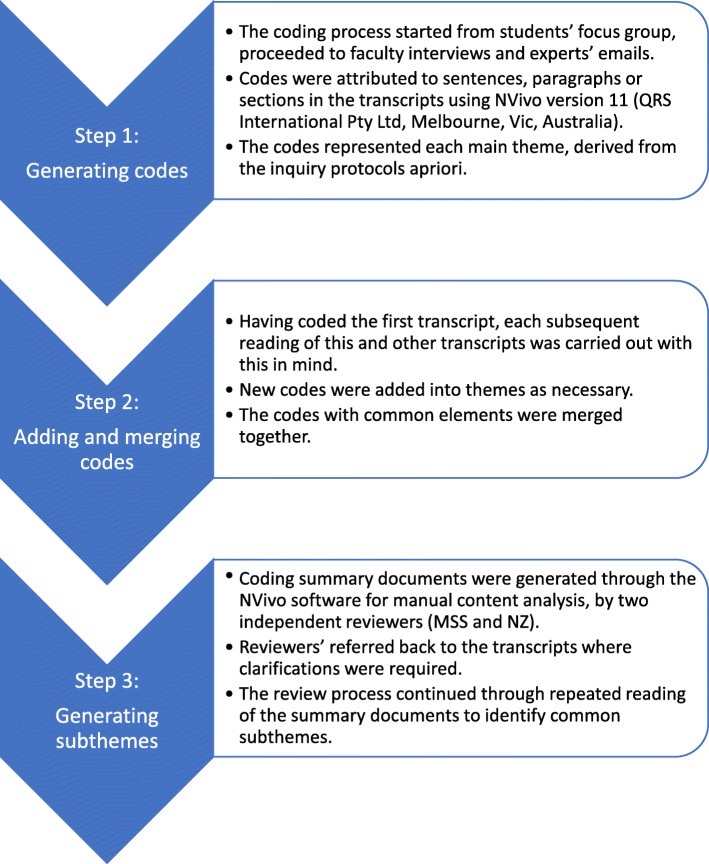
Analysis process

The transcripts were analysed independently by two researchers (MSS and NZ) using NVivo software for coding into the main themes. This analysis informed the coding framework, agreed upon after the consensus of the research team. The framework was then used for driving and documenting inferences from the data on the strengths, weaknesses and areas for improvement, that can be used for refinement of the ethics course and the Workbook. The documents were then reviewed manually, with repeated readings, to understand the views of participants and develop subthemes [24, 25]. This deductive method of analysis enhanced the researchers’ understanding of the views of each participant and influenced the systematic development and refinement of the Workbook [24]. Figure 2 illustrates the process for generating codes, themes and subthemes in this analysis. As the collected data from students, faculty and experts were analysed, comparison between data sets was undertaken to inform interpretation [36, 37].

## Results

The analysis of qualitative data generated twenty-one sub-themes within the four main themes: design features, content, teaching methods and assessment exercises in the Workbook (Table 2). The views of the participants are described under each main theme. The description allows the themes to be viewed through the lenses of each group of participants. The language of quotes is minimally corrected for better understanding while maintaining the authenticity of what was said.

**Table 2:** Content analysis framework

Main Themes/ Categories	Sub-themes generated from coding						
**Design features**	User-friendly & enjoyable (S,F)	Facilitates understanding (S,F)	Active learning (stimulating critical thinking) (S,F)	Integration of teaching methods (S,F,E)	Internalization of learning (S,E)	Language and flow (F,E)	Sustainability
**Content**	Topics covered (F,E)		Size (S,F,E)	Depth of information (F,E)		Contextuality (S,F,E)	
**Teaching methods**	Reflection (S,F,E)	Role play (S,F)	Case discussions & experience sharing (S,F)	Videos (S,F)	Feedback (S,F)	Student presentations (S,F)	
**Assessment exercises**	Quantity (S,F,E)		Amount of information (S,F,E)	Level of difficulty (S,F)		Relevance and contextuality (S,F,E)	

*S* students, *F* faculty, *E* experts

### Theme 1: design features

The students reflected on their learning experience during the workbook-based ethics course and compared it with their previous experience of didactic teaching of ethics. Participants credited the design of Workbook as the main strength of the course in making it an enjoyable and engaging learning experience. They attributed this to the method of course delivery through the combination of the content of the Workbook, including exercises and activities, and various teaching methods. They believed the course influenced their way of thinking and reflection of ethical issues in the hospitals.

*“I have learned that it was wrong what I was thinking … it (the course) has changed the way I think” (FG 1); “What you learn, you have to write it in the workbook, and when you write things, you remember better” (FG 3)*

The experts in their feedback also recognised this potential ability of the Workbook for making the learning experience active in nature and influencing the students’ thought process. They predicted that moral experiences through learning activities and ethical decision-making exercises within the Workbook could modify the way students think.

*“It is not about information, but about the process of thinking in reflection and how they can anticipate the consequences for each option in decision making” (E201 medical educationist, Saudi Arabia); “(The workbook supports) competence development appropriately for the level of education … providing moral experiences” (E 501 ethics teacher, Mexico)*

The experts among the respondents generally considered the design features appropriate for ethics course delivery. However, they identified areas for improvement by suggesting to remove specific topics from the Workbook, enhance the integration of teaching methods with assessment exercises and highlighted the need for a teachers’ guide to using the Workbook. They indicated that the Workbook should be a dynamic document, with potential for modifications as per the requirements of teachers and students.

*“Facilitates effective and active learning … (encourages) students to think and develop ideas … combines theory with practice” (E 1001 ethics teacher, Turkey); “broad range of issues that lend themselves to active, practical examples that can challenge the students in terms of their decisions” (E 301, Canada)*

Faculty members’ experience in undergraduate education in the region was reflected in their views and suggestions regarding the Workbook. They considered the Workbook to be aligned with “*adult learning*”, “*self-directed learning and guided learning*” principles. They expressed their satisfaction with the overall design features of the Workbook as the basis for enhancing students’ interest and motivation during the course.

*“Those (students) who were always dormant started coming out of their shells, and we felt that with the help of this (work) book they even started interacting verbally as well, and therefore they become a part of the whole discussion.” (FI 2).*

Like the experts, they also suggested combining different teaching methods to enhance students’ learning and make it more effective.

### Theme 2: content

The critical review of the content of the Workbook was evident by the constructive criticism by faculty and experts on various sections. Most of them considered the section on *“controversies in contemporary ethics”* in the Workbook as too complicated for the level of student ability. For example, one of the experts opined that such complex issues in the content might cause “*cognitive overload”* for the students. They suggested replacing this section with topics that are more relevant to the level of undergraduate students.

*“ … content at times is too sophisticated and too extensive for such a target audience” (E 101 ethics teacher, Pakistan)*

Participants emphasised that faculty and students should have a clear understanding of the objectives of different activities; therefore, learning objectives should be clearly stated with every topic and activity. Faculty and experts also noted the lack of guidance for facilitators within the Workbook. They mentioned that, for most topics, the facilitator has to bring in contextuality in the discourse. They suggested adding guidance for facilitators in the form of contextually relevant examples that are aligned with the course objectives. This guidance will help the facilitator in generating discussions during the course.

Regarding the content of the Workbook and topics discussed within the course, students expressed a range of views. On the one hand, the majority appreciated that most of the topics in the Workbook are relevant to problems that they may have to face as professionals. On the other hand, some students pointed out that there were only “*few things about religion and culture*” in the course and requested more content from local cultural, religious and legal perspectives to enhance relevance.

*“(Workbook should contain more on) the principle of ethics, and the ethics of Islam as a religion because this is an Islamic country, so that gives them interest as well, and relatedness as well”. (FI 4)*

These responses reflected the socio-cultural structure of the region. However, such views were not unanimous, as others disagreed and supported the view that medical ethics should be taught independent of religion. These students believed that patients could be from various belief systems and cultures, but they must be treated in the same “*standard ethical manner*”.

*“ … in every country they have different religions and different social issues and social problems. Like here in Saudi Arabia, the woman must wear hijab and male doctor, or male student cannot examine a woman easily. This is very (big) problem in Saudi Arabia, but outside it’s not that (big a) problem, so the ethics (course) or Workbook must contain the two ideas” (FG 2)*

This observation was also seen in the responses from some of the experts. The experts from within the region recommended adding information regarding the Islamic (clerical) rulings on various aspects of healthcare issues, doctor-patient interaction, and differences (and similarities) between ethics of different religions. They endorsed that the content should reflect the differences between eastern and western cultures while giving due attention to local perceptions and sensitivities in using the terminologies like *“God”* and *“Messiah”*, which are perceived differently in the local context.

*“Use the designation Allah rather than God in the document (Workbook). Exemplifying healers as the “Messiah prophet” is a controversial issue (in the region).” (E601 medical educationist, Saudi Arabia)*

### Theme 3: teaching methods

Students considered teaching methods, like videos and role-plays, as an integral part of the course that gave them *“experience of real situations”* and recommended more connection between them and reflective writing exercises. The videos were considered supportive for understanding issues and useful in creating a favourable learning environment. Students appreciated the individual feedback, which was given to them during the course on their reflective writing assignments.

*“The process in the book was very good, and the content of the workbook was applied by videos and other methods … made everything clear and helped form my point of view” (FG 2).*

One of the main weaknesses identified by students was related to student presentations. Students expressed their dissatisfaction regarding the topics given for presentations and questioned its benefits. They suggested that group presentations should be replaced with more small group discussions and reflective writing; *“Presentation is good for the one who is making (it)” (FG 1)*.

### Theme 4: assessment exercises

Students reflected that incorporation of assessment within the Workbook, through assignments and classroom quizzes conferred to them a *“sense of freedom”* from end-of-course exams and reduced the stress related to the course. However, one of the focus group participants pointed out that some students may not take the course seriously because there was no exam at the end. In his opinion, it may cause a potential problem in future courses.

Faculty members considered design of assignment exercises to be *“thought-provoking”* and “*constructivist*” in nature, thereby making students responsible for their learning, and engaging students’ interest and active participation.

The experts appreciated the assignment exercises. The ethics educationists found them *“adequate”, “useful”,* and *“creative and diverse”*. However, they suggested to improve them by adding relevant topics in the case scenarios and assignments, like social issues, medico-legal laws and regulations in the country.

*“The scenarios (vignettes) need to be more structured and ill-defined, as in real clinical practice. The students may not have enough background knowledge from practice or legal orientation on how to deal with difficult cases like in Scenario 1 & 2, related to DNR. Student learning can be enhanced by providing options for decision making, and teacher should be ready to discuss the consequences of each one” (E201 medical educationist, Saudi Arabia).*

## Discussion

This study critically examined and refined the preliminary version of the WBEL strategy by analysing the data gathered from a range of stakeholders, including students, course faculty and external experts. The multiple sources of data enhanced the rigour of the research and enhanced the value of the findings [21, 22].

Faculty members from diverse educational and cultural backgrounds (Table 1) reflected on their personal experience of using the strategy in the ethics course. Their critical observations about the effects on students informed comprehension of evidence gathered from students’ themselves [35]. The external experts, on the other hand, mainly reflected on the design, content and possible influence of the strategy on the learners. It was evident that among these experts, those with a background in medical education reviewed the Workbook from an educational perspective. Their primary concerns related to the possible influences on the process of learning. At the same time, the experts with a background in teaching ethics focused on how it may impact on learners’ ethical development. This combination and mixed backgrounds of study participants injected diversity into the research findings, which was particularly beneficial for the refining process of the educational strategy.

The cognitive load in a learning experience is considered a significant factor affecting students’ ability to learn [38]. This was noted by the study participants who contemplated the size and depth of information in the Workbook to be appropriate (minimal extraneous load); the content to be *“easy to understand”* and *“enjoyable”* (enhanced intrinsic load); and the reading material and exercises to be thought-provoking (optimised germane load). It was also apparent from the findings that any significant change in the cognitive load will not be favourable, and the refinement process should enhance the educational potential of WBEL without increasing the cognitive load for students.

### How the process refined the strategy

The WEBL strategy was iteratively refined during this study (Table 3). Efforts were made to enhance the identified strengths and address the issues identified for attention by the research participants. New sections were added to the Workbook dealing with the history of development of medical ethics, local and international laws governing the ethical conduct of healthcare providers and religious and socio-cultural factors relevant to the care of patients. These additions encompassed relevance, augmenting the contextuality in the strategy. Similarly, the pre-existing sections were reinforced with the addition of learning objectives, and new contextually relevant case scenarios, reflective exercises and video clips where suggested by the study participants [15, 39].

**Table 3:** Summary of suggestions from the data

Suggested additions	Data source	Action taken
**Guiding the facilitators in conducting the course**		
Guidelines for new facilitators	(FI2 & FI3) (E201 & E301)	Facilitator guide developed and included in the EWB
Use of cases scenarios that explicitly represent opposing viewpoints	(E601)	Added in EWB and facilitator guide
Summary or take-home message	(FG1 & FG3) (FI1)	Incorporated in facilitator guide
**History and philosophy of ethics**		
Development of bioethics principles and their application in medical profession	(E501, E801)	Section added in EWB
**Differing perspectives from legal and cultural aspects**		
Local and international laws related to medical profession	(FG1, FG2 & FG3)	Excerpts from local codes and guidelines added in different sections of EWB
Sensitive issues related to women’s health in local culture and law	(FG2 & FG3)	
More on Islamic perspective in local culture, Islamic rules from Hadith and comparison of Islamic ethics with ethics of other religions	(FI1) (E701 &E1201)	Section on Islamic ethics in EWB enhanced
More on euthanasia to understand the two sides of views	(E601)	
Identifying and managing presuppositions and biases, and understanding opposing views on different issues	(E301, E601 & E1001)	Added in EWB and facilitator guide
Concept of privacy	(E101 & E1201)	Added in facilitator guide for discussion
**Interactive learning activities**		
More feedback, role-plays and videos	(FG1, FG2 & FG3) (FI1)	Added in EWB
Reflective writing exercises should be added to video clips and role-plays	(FG2 & FG3) (FI4)	Incorporated in EWB
More to enhance students’ communication skills	(FG1 & FG2) (FI3)	Added in EWB and facilitator guide
Links to learning resources	(E601)	Added in EWB and facilitator guide
**Clarity of goals to achieve through different activities**		
Message to be taken from each session	(FG1 & FG3) (FI1) (E601)	Constructive alignment of the EWB enhanced and table added in EWB
Learning objectives with video clips and role-plays	(FG2 & FG3) (FI4)	Added in EWB
**Suggested deletions**	**Data source**	**Action taken**
Repetition in the consent part	(FI4)	Corrected
Avoid the use of culturally controversial words like “Mesiah”	(E601)	Deleted from EWB
Common controversies in last section is not required at this level	(FI2) (E101 & E201)	Deleted from EWB
Prenatal issues and fetal rights are not required at this level	(E101)	Deleted from EWB
Student presentations were not effective for learning of all students	(FG1, FG2 & FG3)	Removed from the course

*FG* students focus group, *FI* faculty interview, *E* experts’ opinion, *EWB* Ethics Workbook

In this study, students acknowledged that the process and tools used for delivery of the ethics course were the main reason for their motivation to learn and internalise the new knowledge. They noted that internalisation (and change in their thinking) was achieved through a combination of short readings from the Workbook, along with interactions with facilitators and peers during lectures and group discussions; followed by reflective writing exercises and feedback. All of these combined as “authentic learning activities” [40] for transforming knowledge from abstract to useful and applicable, thereby modifying students’ ways of thinking.

Study participants also valued other teaching methods used within the ethics course. These methods, including the case discussions, demonstrations, and role-play exercises, were used to supplement the preliminary WBEL strategy during the course [13]. These provided the students with opportunities to experience ethical dilemma in an open and safe environment [41]. The students and course faculty suggested that supplementary methods should also be incorporated as part of the WBEL strategy, along with descriptions and exercises in the form of planned authentic learning activities.

How ethics should be assessed is an ongoing debate in medical education literature [7, 42, 43]. Although Glick [7] suggests that examination gives additional stimulation to students, Mattick and Bligh [42] argue that it does not ensure learning in the context of ethics. In this study, students’ responses regarding assessment in the course were remarkable. Students considered the assessment exercises in the Workbook as an effective method that supported learning, providing a *“sense of freedom”* because there was no end-of-course exam. However, they were mindful that to complete the assessment exercises within the Workbook, they needed to be attentive in the classroom. Similarly, faculty considered this as using the adult learning principles to their best. The Workbook-based strategy ensured consistency and contextuality in the approach to assessment [44].

The faculty participants in this study had limited prior experience in teaching medical ethics. They showed initial reluctance to facilitate, which was alleviated to some degree after going through the Workbook. The Workbook provided them with knowledge on the topics they were required to teach and guidance on how to facilitate their sessions with students. Experts supported this view by suggesting that the WBEL should guide teachers in delivering ethics education effectively. These findings suggested that to be used as a sustainable and effective strategy; the refined WBEL strategy must contain guidance and support for facilitators of the ethics course. To this end, a topic-wise guide for facilitators was developed and incorporated into the Workbook. The guide added the support required for teaching faculty and ensured more consistency in the delivery of WBEL.

The consultative approach to developing a locally relevant learning strategy that was employed in this study can be utilised for various educational purposes. This study specifically applied the method to the socio-cultural context of Saudi Arabia to develop a contextually relevant educational strategy for medical ethics. With an increasing number of medical institutes in the Middle East and South Asian regions [45], broader applicability of this approach can be explored in countries with similar contexts.

The WBEL, hence developed and refined in this study, is a new strategy for medical ethics education in regions which experience a dearth of contextually relevant guidelines, culturally pertinent literature and trained ethics educators. It is a student-centred strategy to teach ethics in undergraduate medicine, using the Workbook as a structured resource. The strategy is grounded in the framework of contextually relevant ethics education model [14]. The model (Fig. 1) incorporates students’ environments, contexts, experiences, reflections and feedback, and addresses the requirements of medical ethics education in various socio-cultural contexts. The WBEL strategy can guide the process of educational delivery in various curricular types, e.g., as a stand-alone course in a discipline or system-based curricula, or an integrated course in an inter-professional curriculum. The process is guided by an updated version of the Workbook as a resource for students and faculty, containing culturally relevant readings on various medical ethics topics, learning activities like role-plays and student presentations and writing assignments, along with opportunities for reflection and feedback. The Workbook facilitates students’ active contextual learning and guides the “non-ethics-trained” teachers in the delivery of ethics education. The WBEL thereby explicitly addresses the factors that limit the delivery of contextually relevant ethics education in the region.

### Limitations

The study participants included a relatively small sample of students due to small class size and potential language issues. The single gender among the participating students was a limitation that was beyond the researchers’ control as the institute only had male students at the time of the study. Additionally, the small number of 3 to 5 students in focus groups is not optimal for generating discussions. This could be enhanced with more than five students per group. The authors expect that triangulation of data from multiple sources may have minimised the effects of these limitations to some extent [28, 46].

The relationship of the researcher to the study participants can play a role in qualitative research [31, 32]. Two of the researchers in this study, MSS and NZ, were faculty members at the RFM, KAU. Their relationship with the participants and awareness of the educational environment may have helped them to understand the issues discussed during data collection and interpret the findings. To ensure rigour in this study, the researcher (MSS) discussed these issues upfront with the participants and informed them about the steps taken to safeguard their privacy and confidentiality. Nevertheless, this relationship can impact on the process; hence the possibility of research bias needs to be acknowledged.

## Conclusion

The 3-pronged consultative approach adopted in this study enabled the identification of key features to iteratively enhance the delivery of medical ethics education through the WBEL strategy. The use of the preliminary version of the Workbook as a trigger enabled a structured investigation of diverse views from various stakeholders in the studied region. These participants identified relevant issues related to the themes of design, content, teaching methods and assessment exercises. They highlighted the importance of well-established principles, such as constructive alignment and contextual relevance. The impact of this strategy on ethics education in the given socio-cultural context will be measured in a later project.

This study contributes to the literature in many ways. The context-specific outcomes of this study are likely to be useful to other educators who are developing educational resources to guide ethics education in similar settings. The process that is reported in this manuscript may be more broadly relevant to guide contextually relevant, evidence-based educational design.

## Supplementary Information

**Additional file 1.**

## Data Availability

All the data from this study is submitted to the UNSW Compactus as per the University policy for safeguarding and future retrieval. The data that support the findings of this study are available from UNSW, but restrictions apply to the availability of these data as they are not publicly available. Data are however available from the authors, MSS and CB, upon reasonable request and with permission of UNSW.

## Abbreviations

WBEL: Workbook based ethics learning
RFM: Rabigh faculty of medicine
FGD: Focus group discussion
IDI: In-depth interview
KAU: King Abdulaziz University
UNSW: University of New South Wales
MSS: Muhammad Shahid Shamim
NZ: Nadeem Zubairi
